# A Novel Tumor-Associated Neutrophil-Related Risk Signature Based on Single-Cell and Bulk RNA-Sequencing Analyses Predicts the Prognosis and Immune Landscape of Breast Cancer

**DOI:** 10.7150/jca.100338

**Published:** 2024-09-03

**Authors:** Shulei Yin, Chunzhen Li, Yunyan Zhang, Haofeng Yin, Zhezhe Fan, Xibo Ye, Han Hu, Tianliang Li

**Affiliations:** 1 National Key Laboratory of Immunity & Inflammation, Institute of Immunology, Naval Medical University, Shanghai 200433, China.; 2 Department of Respiratory and Critical Care Medicine, Changzheng Hospital, Naval Medical University, Shanghai 200433, China.

**Keywords:** breast cancer, tumor-associated neutrophils, risk assessment, tumor microenvironment, immunotherapy

## Abstract

Tumor-associated neutrophils (TANs) are increasingly recognized as contributors to cancer prognosis and therapeutics. However, TAN-related targets of breast cancer (BRCA) remain scarce. This study aimed to develop a novel TAN-associated risk signature (TANRS) of BRCA using single-cell RNA sequencing (scRNA-seq) and bulk RNA sequencing data. Eighty-six TAN-related genes (TANRGs) were derived from the intersection of TAN marker genes identified from scRNA-seq with modular genes identified by weighted gene co-expression network analysis (WGCNA). The TANRS consisting of nine TANRGs (TAGLN2, IGF2R, LAMP2, TBL1X, ASAP1, DENND5A, SNRK, BCL3, and CEBPD) was constructed using Cox regression and the least absolute shrinkage and selection operator (LASSO) regression. The TANRS efficiently predicted the survival prognosis and clinicopathological progression of patients across multiple cohorts. Significant differences in immune infiltration landscapes between TANRS groups were observed. Additionally, patients with high TANRS exhibited tumor immunosuppression, enhanced cancer hallmarks, and unfavorable therapeutic effects. Four promising compounds for treating high-TANRS BRCA were also presented. SNRK was identified as a key prognostic TANRG, and its expression profile and correlation with TANs were validated using immunohistochemical assays of BRCA samples and spatial transcriptomic sections. This novel TAN-based signature exhibited promising predictive capabilities, with the potential to contribute to personalized medicine for BRCA patients.

## Introduction

Breast cancer (BRCA) is the second most widespread and fatal malignancy in females, and its mortality rate continues to rise annually 1,2. In 2020, there were an estimated 2.3 million new cases of BRCA worldwide, resulting in approximately 685,000 deaths 3,4. BRCA is a heterogeneous disease characterized by diverse biological phenotypes, specific histologic features, variable clinical manifestations, and therapeutic vulnerabilities 5,6. Despite significant advancements in our understanding of the biological function, molecular and cellular mechanisms, as well as diagnosis and treatment approaches for BRCA, the prognosis remains bleak due to tumor metastasis, recurrence, and inadequate treatment response 5,7,8. Furthermore, after the diagnosis of breast cancer, the immediate challenge in patient management lies in determining the prognosis and selecting the most appropriate systemic therapy. The most reliable approach to assessing the prognosis involves considering traditional clinical prognostic factors, biomarkers (such as HER2/neu), and specific multi-gene tests 9. However, the current availability of reliable tools for predicting BRCA patient outcomes is limited. Therefore, discovering new biomarkers to efficiently evaluate the patient prognosis and therapeutic outcome is critically required.

The infiltrative immune cells in the tumor microenvironment (TME), including T cells, NK cells, macrophages, and dendritic cells (DCs), have been identified as crucial factors influencing prognosis and immune invasion among patients with BRCA 10-12. Among these cells, tumor-associated neutrophils (TANs) are believed to possess immunosuppressive properties and exacerbate patient outcomes 13,14. The pro-tumor phenotype is characterized by promoting cell proliferation, angiogenesis, and immunosuppression within the TME 15,16. Recent studies have highlighted the increasing significance of TANs in exerting anti-tumor effects during tumor progression and immunotherapy 17-20. These contradictory data raise critical but poorly understood questions regarding the role of TANs in the pathogenesis and progression of BRCA. Therefore, conducting an exhaustive investigation into the functional role of TANs and identification of a prognostic signature associated with TANs in BRCA patients are imperative and scientifically sound approaches.

**Figure 1:**
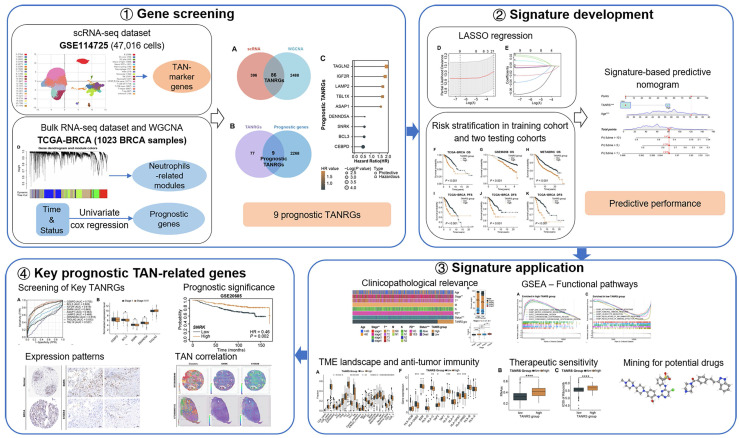
The workflow illustration of this study.

In this study, we performed a comprehensive analysis to identify novel TANRGs associated with BRCA prognosis. Subsequently, we developed a new TAN-related gene signature, whose prognostic performance and clinical association were demonstrated in multiple cohorts. Furthermore, the signature functioned well in analyzing the tumor microenvironment landscape, immune function, and therapeutic vulnerabilities while also predicting potential anticancer drugs. An overview of our study protocol is illustrated in **Figure 1**.

## Materials and methods

### Acquisition and processing of data

Bulk RNA-seq data for 1109 BRCA samples and 113 normal samples, together with their clinical details, were downloaded from the Cancer Genome Atlas (TCGA) database (https://portal.gdc.cancer.gov/). Duplicates and cases with less than 60 days of overall survival (OS) were deleted to exclude very early deaths due to specific causes and ensure the accuracy of the long-term prognostic analysis, resulting in 1023 BRCA samples for further analysis. The scRNA-seq dataset (GSE114725) of samples from 8 BRCA patients was obtained from the IMMUcan database, containing 47016 BRCA-infiltrating immune cells 21,22. Single-cell data processing and analyses were done based on the IMMUcan database. According to the official instructions and the dataset description, the data were preprocessed using the pipeline called SEQC, and cells containing more than 25% of mitochondrial reads were excluded 22. Cell types were identified by integrating the CHETAH algorithm embedded in the database and annotations provided by the original authors of the dataset. Visualization processes were done through specific modules of the IMMUcan database, using the official default settings. Transcriptomic and clinical data from two independent BRCA cohorts, GSE96058 (N=3273) and METABRIC (N=1896), were applied for external validation.

### Assessment of TAN abundance and survival analysis

The abundance data for tumor-infiltrating immune cells (TIICs) in TCGA-BRCA samples were downloaded from the TIMER2.0 database 23. The MCPcounter algorithm was employed to quantitatively determine the abundance of TANs for further classification 24. The optimal cut-off value of TAN abundance for Kaplan-Meier survival analysis was determined through survminer and survival R software packages.

### Acquisition of prognostic TANRGs

TANRGs were identified through a combination of scRNA-seq and bulk transcriptomic WGCNA analyses. TAN marker genes were obtained from differential analysis of cell types. WGCNA procedure was applied to identify gene modules with the most correlation to neutrophil infiltration. Then, the optimal soft threshold is determined for efficient operation in WGCNA. Reliable TANRGs were further screened by intersecting genes derived from the most relevant WGCNA modules with TAN differential genes obtained from scRNA-seq data. Prognostic TANRGs were further yielded by univariate Cox analysis using the "survival" package.

### Construction and validation of a TAN-related risk signature (TANRS)

By conducting multivariate Cox and LASSO regression analyses on prognostic TANRGs within the TCGA-BRCA cohort, we identify the optimal TANRGs and their corresponding coefficients for constructing a TANRS. To assign TANRS to each sample, we employed the following formula:

TANRS = 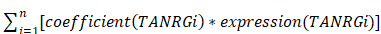


Then, patients were stratified into high-TANRS and low-TANRS groups according to the optimal TANRS to fully demonstrate its prognostic effects. Survival differences between the two TANRS groups were subsequently compared. The validation cohorts were stratified utilizing the same grouping approach as the TCGA-BRCA cohort.

### Performance evaluation and clinicopathological relevance of the TANRS

The predictive accuracy of TANRS for survival was assessed using time-dependent receiver operating characteristic (ROC) curves and calibration curves with the use of R packages including "rms", "timeROC", "survival" and "survminer". Additionally, we investigated the relationship between TANRS and clinicopathological parameters. The predictive independence of the TANRS was assessed using Cox regression analyses. Finally, independent prognostic clinicopathologic parameters were integrated with TANRS to construct a predictive nomogram.

### Functional pathway enrichment analysis

The Gene set enrichment analysis (GSEA) was performed to identify the pathways associated with this TANRS in BRCA. Gene sets of Gene Ontology (GO) and Kyoto Encyclopedia of Genes and Genomes (KEGG) were downloaded from the MSigDB. With the use of "org.Hs.eg.db" and "clusterProfiler" packages, we could conduct GSEA and obtain the pathways or processes associated with our groups 25. GO or KEGG items that meet the following conditions are considered significantly enriched: |normalized enrichment score (NES)|> 1 and p-value < 0.05.

### Analysis of tumor immune landscape and immunotherapy

To evaluate the immunological relevance of TANRS in BRCA, we quantified the abundance of TIICs and calculated the TME score, tumor purity, and immune function score. Following the official instructions, we uploaded the transcriptomic data to the ImmunecellAI platform and retrieved the results using its TIIC prediction module 26. We utilized Spearman's method to evaluate correlations between the TANRS and levels of TIICs. Additionally, we employed the ESTIMATE algorithm to quantify the immune, stromal, ESTIMATE, and tumor purity scores. To assess immunological characteristics in patients from both TANRS groups, a GSVA-based immune signature scoring system was used. The expression of immune checkpoint molecules and antigen-presentation-related molecules were compared to assess the immunosuppressive and antigen-presentation characteristics of the tumors, which were combined with the IPS score to analyze the vulnerability of the patients to immunotherapy 27.

### Chemosensitivity characterization and candidate compound screening

Cancer stemness is strongly associated with its chemotherapeutic sensitivity, so we first assessed the correlation between TANRS and tumor stemness 28,29. Then the half-maximal inhibitory concentration (IC50) of several anticancer agents was assessed using the "pRRophetic" package 30. This evaluation aimed to compare the sensitivity of these drugs between patient groups with different TANRS levels. Differences in transcriptomics between the two TANRS groups were uploaded to the Connectivity Map (Cmap, https://clue.io/) to explore potential pharmacological interventions for high-TANRS BRCA based on official tutorials 31.

### Screening and validation of key TANRGs

To characterize the TANRGs closely related to the prognosis and progression of BRCA, the association between the expression of these TANRGs and the prognosis and pathological progression of BRCA was evaluated. ROC curves were shown to demonstrate the prognostic performance of signature TANRGs. Additionally, we employed external cohort validation and clinicopathological analyses to further determine the key TANRG 32. We also utilized publicly available immunohistochemical (IHC) images from the Human Protein Atlas (HPA), proteomic data from the Clinical Proteomic Tumor Analysis Consortium (CPTAC) database to confirm the protein-level expression of the key TANRG, SNF1-related kinase (SNRK) 33.

### Tissue microarray-based IHC assay and spatial transcriptome analysis

Following the analysis of the SNRK-TAN correlation based on the TCGA-BRCA dataset, IHC assays based on BRCA tissue chips (purchased from Servicebio, Wuhan, China) were performed to assess the abundance of one of the TAN markers, S100A9, in tumor regions with different SNRK levels. The Spatial transcriptome data from STOmics DB were further employed to validate the correlation between SNRK and S100A9 34. IHC assays were performed using primary antibodies against SNRK (Abcepta AP7249c, Suzhou, China) and S100A9 (Servicebio GB111149-100, Wuhan, China), and the staining procedure was conducted as previously described 35, 36.

### Statistical analysis

Statistical analyses were performed based on R software (4.1.2 version) and online tools. As described in the previous section, multiple R functional packages were applied for data visualization and statistical analyses. The Kaplan-Meier method was employed for survival analysis. Wilcoxon test and t-test were used for comparison of differences between the two groups. One-way ANOVA was used for comparison of data between multiple groups. Correlations between variables were analyzed using the Spearman method. A p-value < 0.05 was considered statistically significant.

## Results

### Identification of TANs marker genes based on scRNA-seq

**Figure 2:**
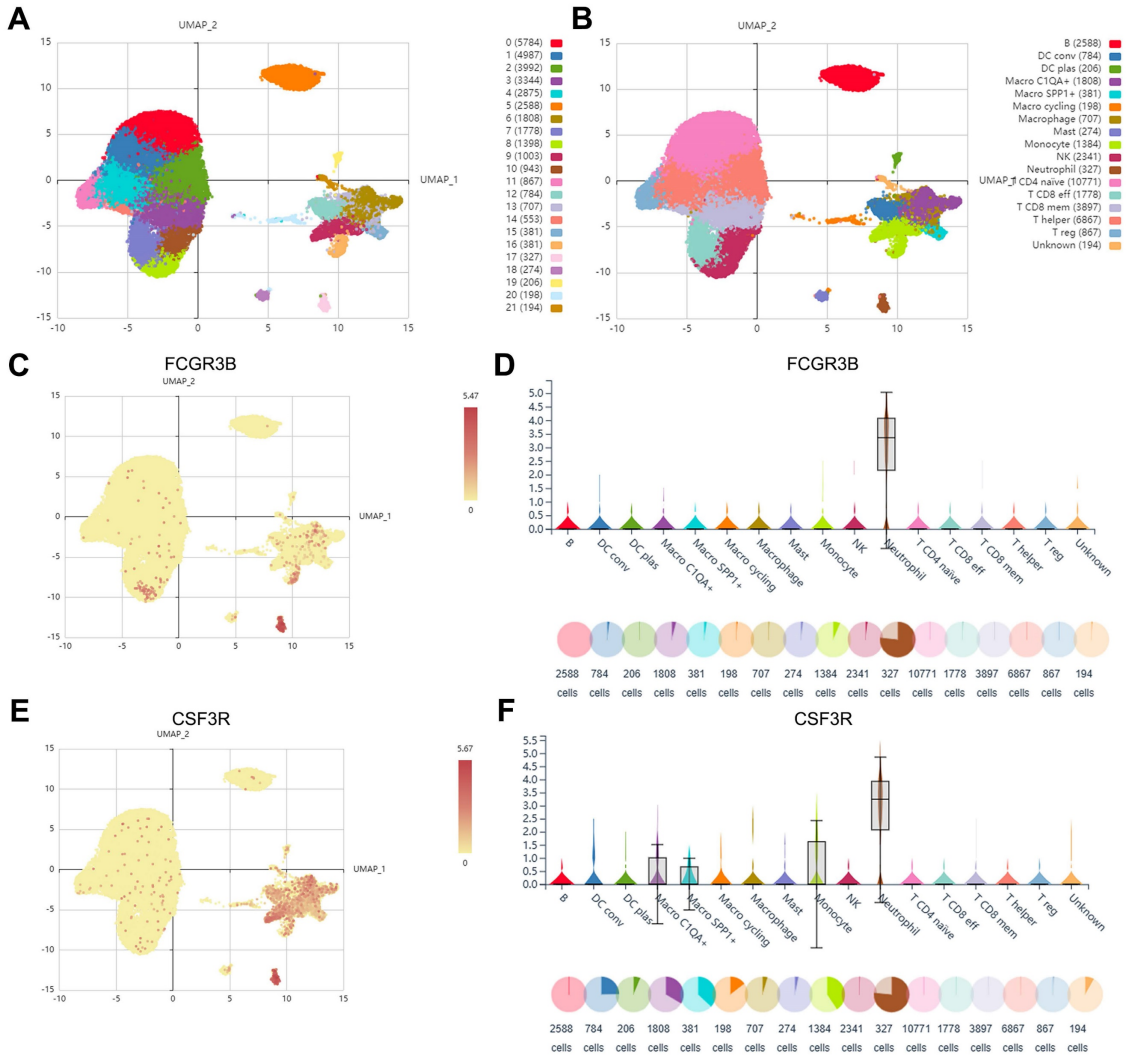
Identification of marker genes of TANs based on scRNA-seq analysis. (A) The Umap plot of 21 cell clusters. (B) The Umap plot of annotated cell types. (C, D) The expression of neutrophil marker gene FCGR3B in different cell types. (E, F) The expression of neutrophil marker gene CSF3R in different cell types.

Based on the inDrop scRNA-seq dataset GSE114725, a total of 47016 enriched immune cells were obtained and grouped into 22 clusters utilizing the Seurat clustering and UMAP plot function on the IMMUCAN platform **(Figure 2A)**. The 22 clusters were further annotated into 17 cell types, including neutrophils, B cells, NK cells, T cell subsets, monocyte-macrophage subsets, DC cell subsets, etc. **(Figure 2B)**. Notably, the cells in cluster 17 were identified as neutrophils with a favorable cluster separation **(Figure 2B)**. Differential expression of marker genes of neutrophils, FCGR3B, CSF3R, and S100A9, was visualized on UMAP plots and violin plots **(Figure 2C-F, Supplementary Figure S1A, B)**. Additionally, cell communication analysis showed that neutrophils exhibit crosstalk with components of the tumor microenvironment, including effector T cells, macrophages, and fibroblasts, fully demonstrating their important effects **(Supplementary Figure S1C)**. In order to initially obtain TAN-related genes (TANRGs) for subsequent analysis, differential analysis of the transcriptomic expression profiles of neutrophils with other cell types was conducted and yielded 482 differentially expressed genes as marker genes of TANs.

### Screening for TANRGs by WGCNA

**Figure 3:**
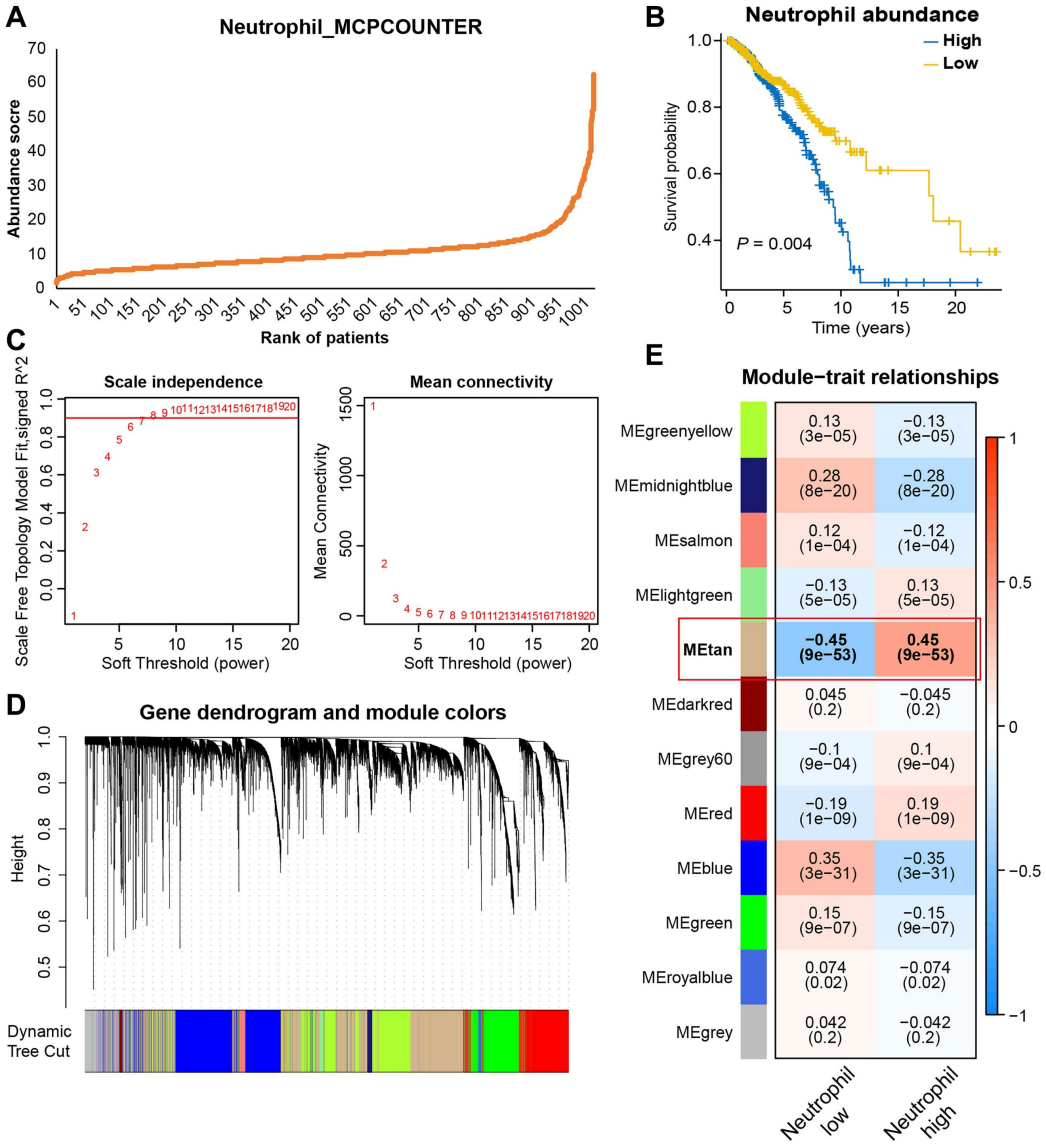
Identifying gene modules most relevant to TAN levels based on bulk transcriptomic analysis. (A) Neutrophils abundance score of BRCA patients estimated by MCPCOUNTER algorithm. (B) Survival analysis based on TAN levels. (C) The best soft-threshold power was identified as 6 in WGCNA. (D, E) Gene clustering and module-trait correlation analysis.

Besides scRNA-seq analysis, we also applied WGCNA analysis to identify genes strongly associated with TAN infiltration based on bulk transcriptomic data. Firstly, the MCPcounter algorithm was used to quantify neutrophil infiltration in the TCGA-BRCA dataset, and every patient was given an abundance score of tumor-infiltrating neutrophils **(Figure 3A)**. Then, patients were divided into high- and low-neutrophil groups. As shown in **Figure 3B**, BRCA patients with high levels of infiltrative TAN exhibited significantly shorter survival, thus suggesting a prognostic role of TAN in BRCA. For WGCNA, seven were selected as the optimal soft threshold for subsequent analyses** (Figure 3C)**. After the clustering of samples and genes, twelve gene modules were identified **(Figure 3D)**, among which the tan-colored module exhibited the strongest and most significant association with neutrophil level (correlation = 0.45, p<0.001; **Figure 3E**). Therefore, 2574 genes in the tan-colored module were collected for the follow-up screening.

### Construction of the TAN-related risk signature (TANRS) using prognostic TANRGs

**Figure 4:**
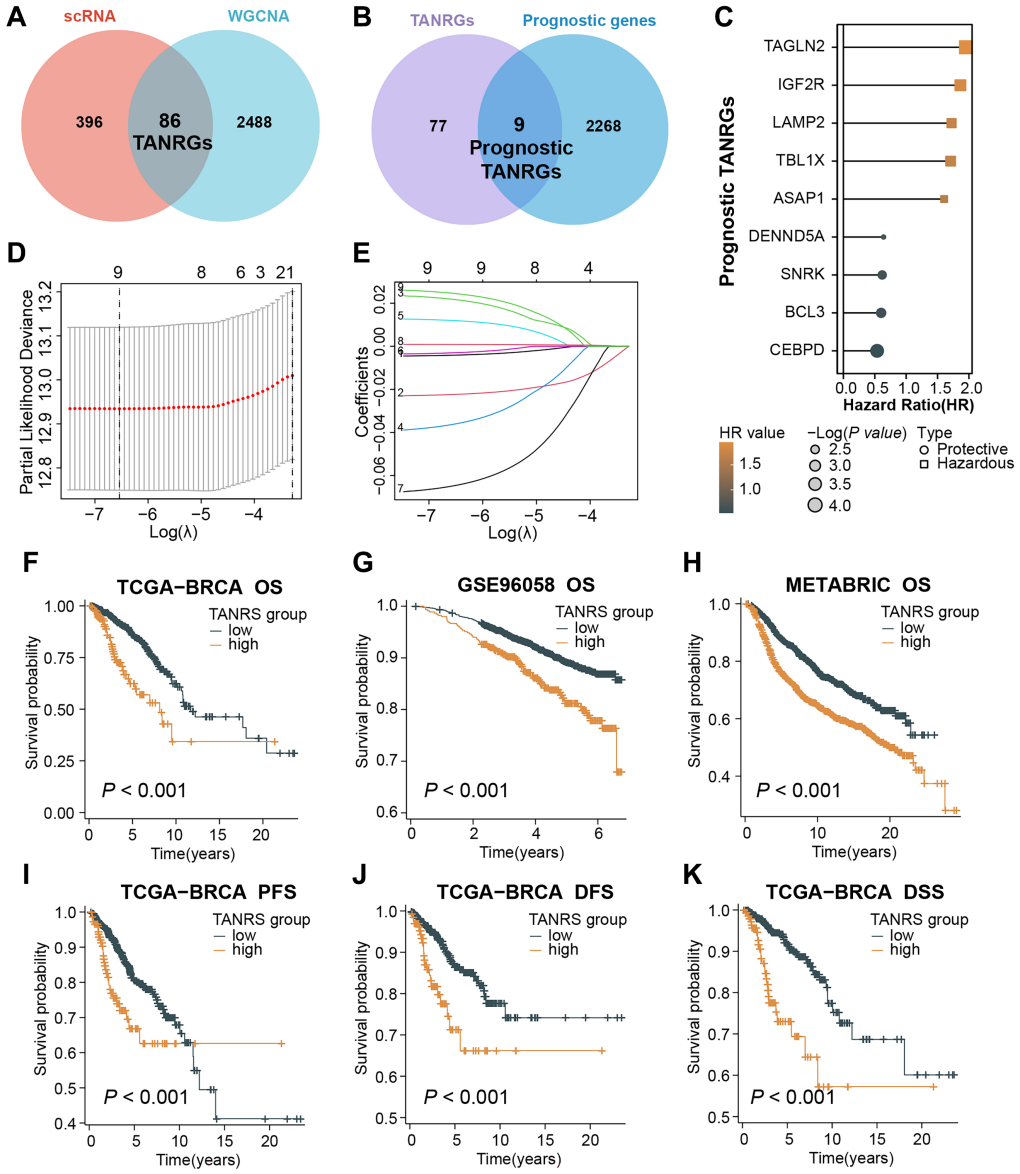
Screening of prognostic TANRGs and establishing the TAN-associated risk signature (TANRS). (A, B) Identification of TANRG by taking stepwise intersections. (C) HR value of nine prognostic TANRGs. (D, E) LASSO analysis confirmed candidate TANRGs and their coefficients. (F-K) Kaplan-Meier curves of BRCA patients with low- and high-TANRS in different cohorts.

After taking the intersection of 482 TAN marker genes derived from scRNA-seq and 2574 tan-colored module genes, we obtained 86 candidate genes considered to be reliable TANRGs **(Figure 4A)**. By further intersecting the 86 candidates with a set of 2277 genes with prognostic value in BRCA, we identified 9 prognostic TANRGs **(Figure 4B)**, including TAGLN2, IGF2R, LAMP2, TBL1X, ASAP1, DENND5A, SNRK, BCL3 and CEBPD. The hazard ratio (HR) values and significance of these TANRGs are shown in **Figure 4C**.

We applied the LASSO regression to determine candidate TANRGs for signature development. Results of LASSO regression suggested that the 9 prognostic TANRGs could be incorporated into the TAN-related risk signature (TANRS), and their corresponding coefficients for calculating signature scores were generated together **(Figure 4D, E)**. Patients in the TCGA-BRCA cohort were divided into low-TANRS and high-TANRS groups according to the best cutoff of TANRS. As expected, patients in the low-TANRS group experienced a better prognosis than those with high TANRS, as evidenced by more prolonged overall survival (OS) (p<0.001; **Figure 4F**). Consistent findings were also observed in validation cohorts, GSE96058 and METABRIC (all p<0.001; **Figure 4G, H**). Moreover, high-TANRS patients also exhibited considerably shorter PFS (Progression-free survival), DFS (Disease-free survival), and DSS (Disease-specific survival) (all p<0.001; **Figure 4I-K**).

### Assessment of the prognostic effect and clinical relevance of the TANRS

**Figure 5:**
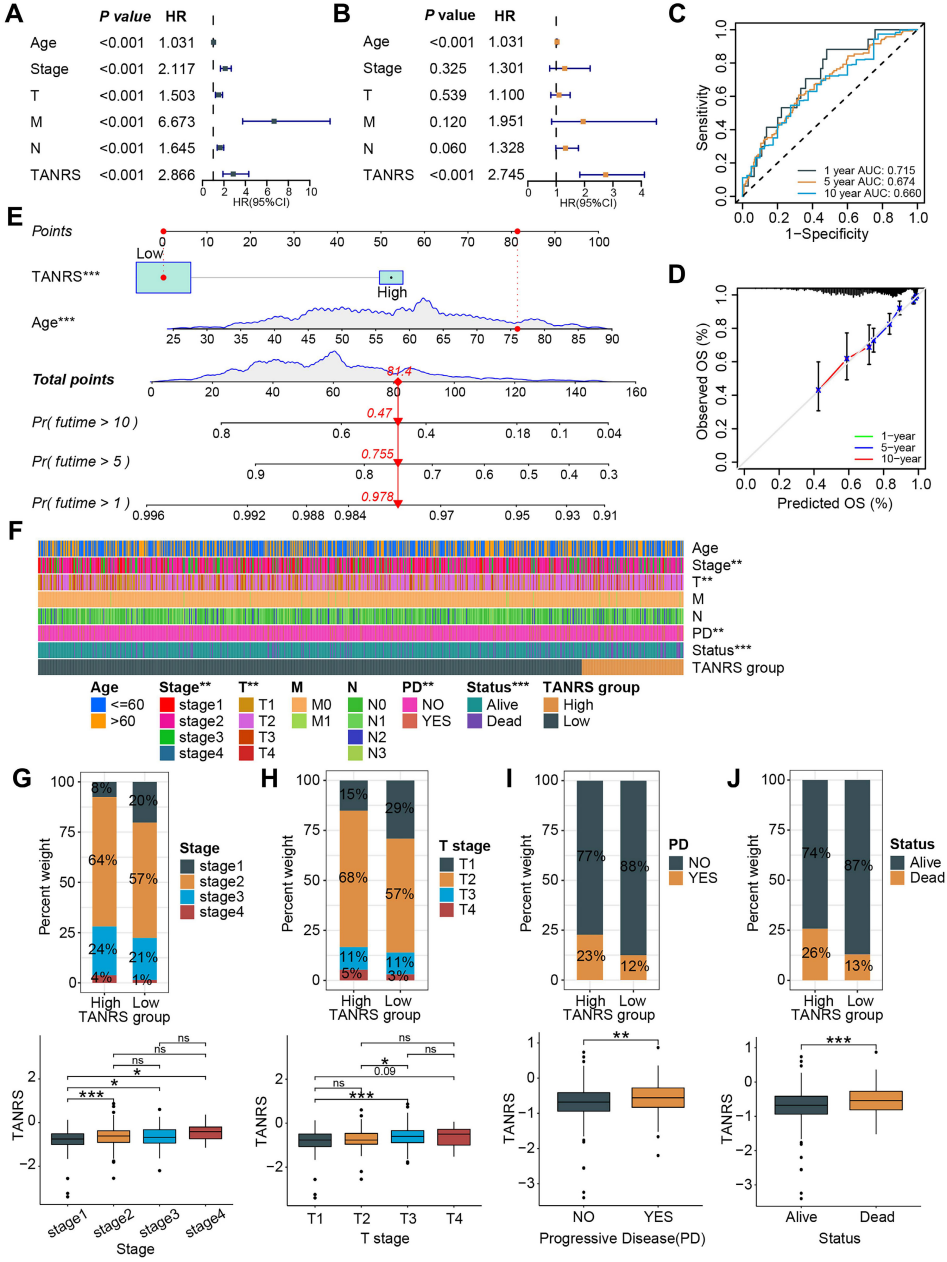
Performance and clinical relevance assessment of the TANRS. (A, B) Univariate and multivariate analyses of the TANRS in the TCGA-BRCA cohort. (C) ROC curves show the predictive performance of TANRS. (D) The calibration curve of the reliable performance of TANRS. (E) TANRS-based nomogram. (F) Heatmap of the distribution of patients with different clinical stages in the TANRS subgroups. (G-J) The correlation between the TANRS and clinicopathological parameters, including stage (G), TNM-T stage (H), disease progression (I), and survival status (J). (ns: not significant, *p < 0.05, ** p < 0.01, *** p < 0.001).

The prognostic value of TANRS in BRCA was further demonstrated through univariate and multivariate regression analyses, establishing it as an independent indicator **(Figure 5A, B and Supplementary Figure S2A, B)**. Time-dependent ROC and calibration curves were plotted to explore the predictive accuracy of the TANRS. The areas under the curve (AUC) at years 1, 5, and 10 were 0.715, 0.674, and 0.660, respectively, which combined with the calibration curves demonstrated the favorable performance of TANRS **(Figure 5C, D)**. Subsequently, we developed a nomogram for accurate and quantitative prediction of overall survival based on this TANRS and age factor **(Figure 5E)**.

The association between the TANRS and clinicopathological indicators such as age, tumor TNM stage, and survival outcome were further analyzed. The heatmap showed that TANRS is associated with tumor stage, T-stage, progressive disease (PD), and survival status **(Figure 5F)**. Specifically, the proportion of patients with stage 2-4 and stage T2-4 tumors in the high-TANRS group was significantly higher than that in the low-TANRS group **(Figure 5G, H).** Furthermore, patients with PD and unfavorable survival outcomes also had significantly higher TANRS **(Figure 5I, J)**. The results above indicated that this TAN-based signature had favorable prognostic performance and clinicopathological relevance.

### The TANRS is closely correlated with immune-related pathways

**Figure 6:**
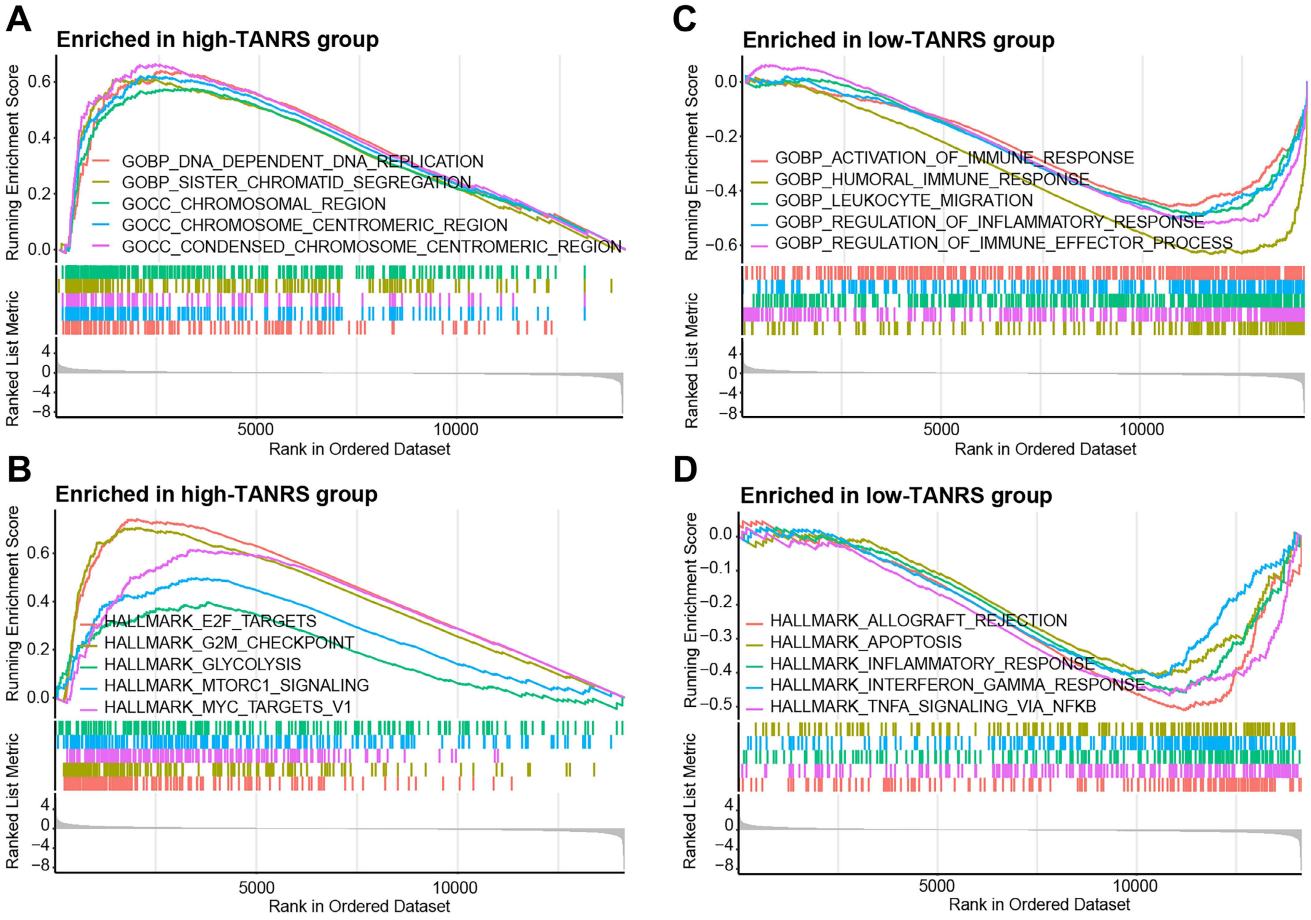
GSEA analysis. Significantly enriched GO terms and HALLMARK pathways in the high-TANRS group (A, B) and the low-TANRS group (C, D).

GSEA was conducted to investigate the potential factors of TANRS leading to prognostic risk and disease progression. High-TANRS tumors were predominantly enriched with pathways associated with DNA replication, chromosomal regions, and E2F targets **(Figure 6A, B)**. Importantly, low-TANRS tumors were strongly linked with immune response, leukocyte migration, interferon-gamma response, and TNF signaling **(Figure 6C, D)**. These findings suggested that tumor immune microenvironment and antitumor immunity may play an important role in determining the differential prognosis of BRCA patients.

### Characterization of tumor immune landscape and immunotherapy

**Figure 7:**
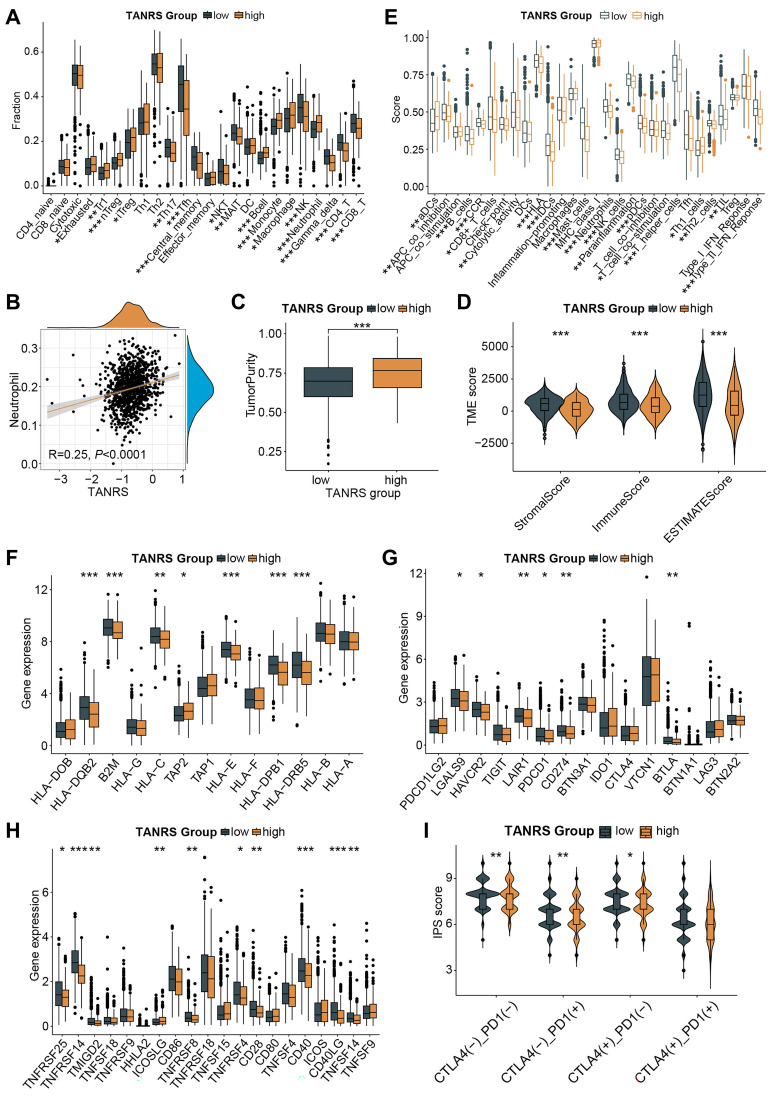
Association between the TANRS and the TME landscape. (A) Analysis of the TIIC abundance. (B) Correlation between TANRS and neutrophil abundance. (C) Correlation of TANRSs with tumor purity. (D) Microenvironmental scores calculated by the ESTIMATE algorithm. (E) Differences in immune cell and immune function scores between high- and low-TANRS groups. (F-H) Expression of antigen processing- and presentation-related genes (F), inhibitory checkpoints (G), and stimulatory checkpoints (H). (I) Differences in IPS scores between high- and low-TANRS groups. (*p < 0.05, **p < 0.01, ***p < 0.001).

To investigate the immune landscape of high- and low-TANRS patients, the abundance of TIICs was analyzed based on the ImmunecellAI platform. Notably, the high-TANRS tumors exhibited more infiltrative exhausted T cells, regulatory T cells (Tregs), monocytes, macrophages, and neutrophils **(Figure 7A)**. While low-TANRS individuals showed increased CD8^+^ T cells, CD4^+^ T cells, helper T cells, memory T cells, NKTs, MALTs, NK cells, and γδT cells **(Figure 7A).** In particular, we confirmed a significant positive association between infiltrative TAN levels and TANRS in BRCA patients, indicating the reliability of this TANRS **(Figure 7B)**. Additionally, compared to high-TANRS individuals, low-TANRS patients exhibited decreased tumor purity and elevated stromal scores, immune scores, and ESTIMATE scores **(Figure 7C, D).** These observations imply that low-TANRS tumors are associated with increased infiltration of immune cells.

We also investigated which immune-related functional phenotypes are affected by TANRS. Obviously, various anti-tumor immune-related functions such as DC function and antigen presentation, CD8^+^ T and NK cell function, interferon response, and cytokines were more potent in patients with low TANRS compared to those with high TANRS, indicating an association between low-TANRS and enhanced anti-tumor immunity **(Figure 7E).** Next, the differential expression of immunomodulatory molecules was analyzed. Tumors with low TANRS expressed higher antigen processing and presentation-related molecules, inhibitory checkpoints, and stimulatory checkpoints **(Figure 7F-H)**. Furthermore, key immunotherapy targets such as PDCD1 and CD274 were found to be downregulated in the high-TANRS group **(Figure 7G)**. Furthermore, low-TANRS tumors were characterized by a higher Immunophenotype Score (IPS), potentially more sensitive to immunotherapy, and were unaffected by the double-negative status of PDCD1 and CTLA4 **(Figure 7I)**. This suggests the reduction of TANRS may also improve the efficacy of tumor immunotherapy through other checkpoint-independent mechanisms, such as altering the landscape of the tumor microenvironment, augmenting antigen presentation and immunogenicity, and enhancing the abundance and cytotoxicity of antitumor effector cells, as supported by the GSEA, GSVA, and TIIC analyses in **Figure 6A-D and Figure 7A-E.** The data suggests that low-TANRS individuals may exhibit a "hot tumor" phenotype, characterized by elevated levels of immune cells within the TME, enhanced immunocompetent effects, and increased tumor immunogenicity.

### TANRS in the prediction of chemotherapeutic sensitivity and potential anti-cancer drugs

**Figure 8:**
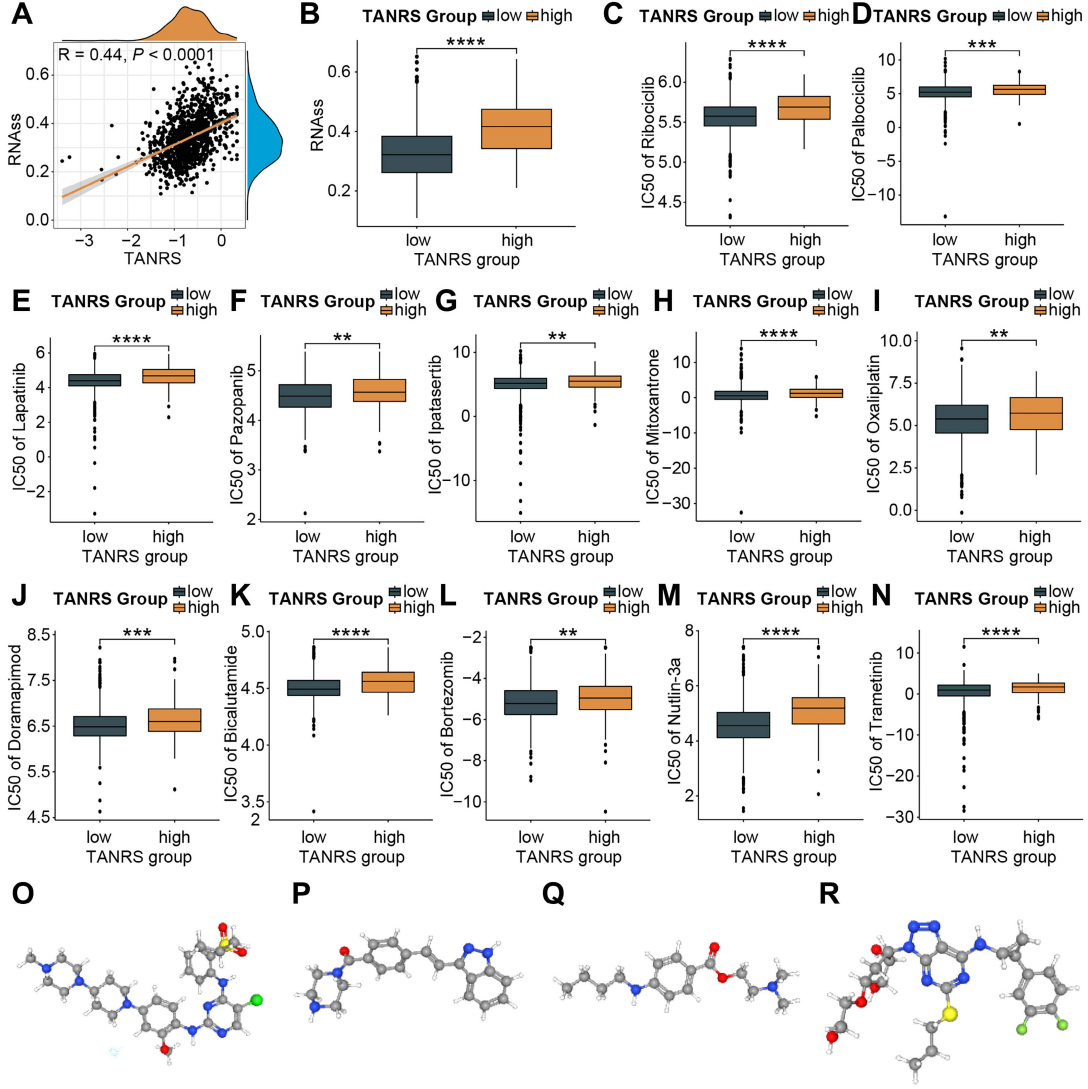
The application of TANRS in guiding clinical chemotherapy sensitivity. (A, B) Correlation between TANRS and tumor stemness. (C-N) IC50 of chemotherapeutic and targeted drugs, including Ribcociclib (C), Palbociclib (D), Lapatinib (E), Pazopannib (F), Ipatasertib (G), Mitoxantrone (H), Oxaliplatin (I), Doramapimod (J), Bicalutamide (K), Bortezomib (L), Nutlin-3a (M), and Trametinib (N) in patients with high- and low-TANRS. (O-R) Structures of four promising compounds (NVP-TAE684, KW-2449, Tetracaine, and Ticagrelor) for the treatment of patients with high TANRS (*p < 0.05, **p < 0.01, ***p < 0.001, ****p < 0.0001).

The association between TANRS and tumor stemness scores was first investigated in light of the enhanced resistance of cancer stem cells to chemotherapy 37. Notably, a positive correlation was observed between TANRS and tumor stemness scores **(Figure 8A, B)**, suggesting the potential and practical use of TANRS in assessing chemotherapy sensitivity. The susceptivity of patients to commonly used chemotherapeutic agents was assessed. High-TANRS individuals exhibited increased IC50 scores for all selected drugs, indicating drug insensitivity and resistance **(Figure 8C-N)**. Thus, low-TANRS individuals may derive greater benefits from these anticancer medications, possibly partly explaining their improved prognosis.

To expand therapeutic options in high-TANRS patients, the Cmap platform was utilized to predict specific molecules that show promise in targeting high-TANRS tumors. NVP-TAE684, KW-2449, Tetracaine, and Ticagrelor were screened as potential compounds suitable for treating high-TANRS patients **(Figure 8O-R)**.

### Identification and validation of SNRK as a key TANRG

**Figure 9:**
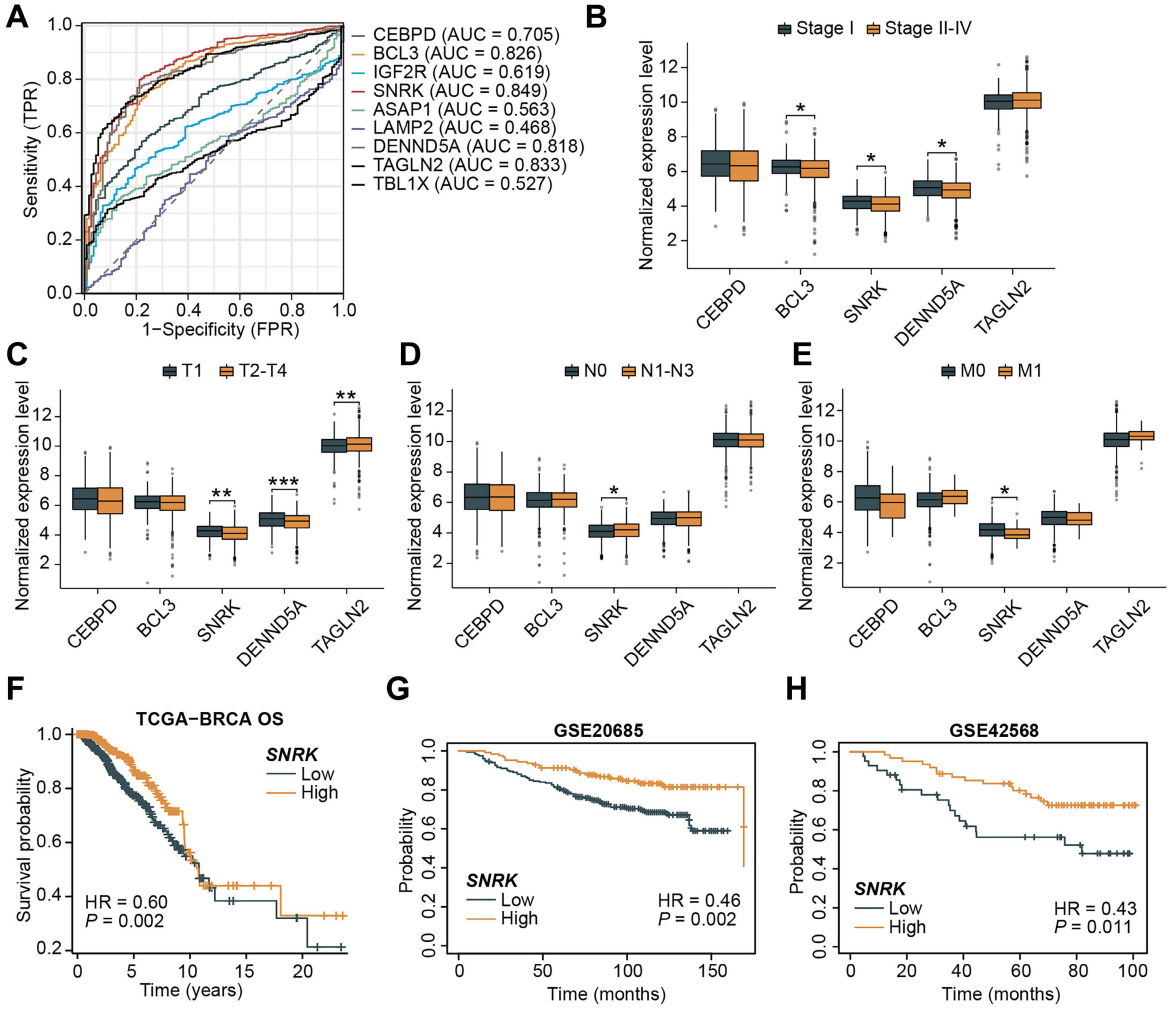
Screening and identification of SNRK as a key TANRG associated with BRCA progression. (A) ROC curves for signature TANRGs and their AUCs. (B-E) Expression of signature genes in BRCA samples in different clinicopathological cases. (F-H) Kaplan-Meier survival curves of high- and low-SRNK patients in TCGA-BRCA and two external cohorts. (*p < 0.05, **p < 0.01, ***p < 0.001).

Aiming to further screen for important TANRGs in BRCA, we assessed the predictive accuracy of TANRGs. As shown in **Figure 9A**, CEBPD, BCL3, SNRK, DENND5A, and TAGLN2 exhibited high sensitivity in BRCA and were identified as key TANRGs for further investigation. Their expression patterns were compared among patients with different tumor stages. Among these five TANRGs, SNRK expression was significantly altered in tumors with different pathological stages. Except for its minor upregulation in the N1-N3 stage, SNRK was obviously downregulated in Stages 2-4, T2-T4, and M1 stages **(Figure 9B-E)**. Therefore, SNRK may be a benign TANRG closely associated with BRCA progression, and survival analyses of the TCGA-BRCA and external cohorts have also demonstrated significantly prolonged OS in BRCA patients with high SNRK expression **(Figure 9F-H)**. High-SNRK patients consistently exhibited longer PFS and DSS **(Supplementary Figure S3A, B)**.

**Figure 10:**
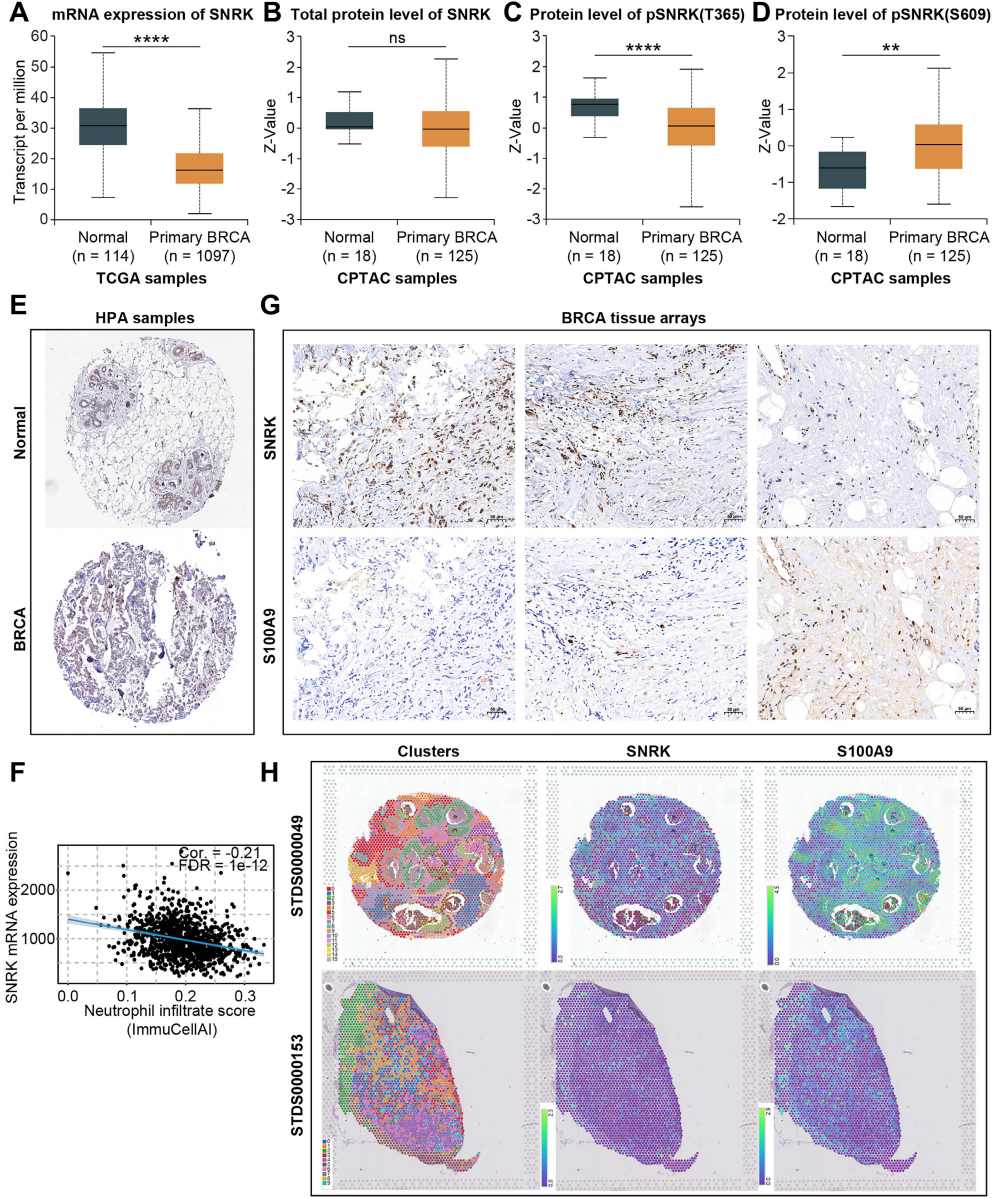
Characterization of SNRK expression in BRCA. (A) RNA expression of SNRK in normal and BRCA tissues in TCGA-BRCA dataset. (B-D) Protein level of toal-SNRK (B), pSNRK(T365) (C), and pSNRK(S609) (D) in normal and BRCA tissues in the CPTAC database. (E) IHC analysis of SNRK in normal and BRCA tissues based on public data. (F) The negative correlation between SNRK expression and neutrophil infiltration score in BRCA tissues. (G, H) Tissue microarray-based IHC assays and spatial transcriptome confirmed the negative correlation between SNRK abundance and the neutrophil marker S100A9 in BRCA tissues. (*p < 0.05, **p < 0.01, ***p < 0.001, ****p < 0.0001).

SNRK (SNF1-related kinase), a serine/threonine kinase first identified from the earliest population of CD34^+^ hematopoietic progenitors that form in the aorta of the human embryo, has been scarcely reported regarding its role in breast cancer and TANs 38. SNRK mRNA expression levels were significantly downregulated in BRCA **(Figure 10A).** Although no significant difference was observed in the total protein levels of SNRK between normal and primary BRCA samples, a notable decrease in pSNRK (T365) expression was detected in BRCA tumor tissues compared to normal tissues, while pSNRK (S609) exhibited higher expression in tumor tissues, suggesting the potential role of SNRK phosphorylation in BRCA **(Figure 10B-D)**. IHC slides based on public databases also confirmed that total protein levels of SNRK were comparable in BRCA and para-cancerous tissues **(Figure 10E, Supplementary Figure S3C)**.

Moreover, bioinformatic analysis indicated that SNRK expression was significantly negatively correlated with neutrophil levels, and we verified this negative correlation using BRCA microarray-based IHC assays and public spatial transcriptomic samples **(Figure 10F-H)**. The level of TAN infiltration was lower in regions with higher SNRK abundance in the sample sections, while the trend was the opposite in low-SNRK regions. These results suggested that SNRK was a key TANRG associated with patient prognosis and TAN infiltration in BRCA.

## Discussion

TME has gained increasing attention regarding its role in prognosis and anti-tumor immunity. The well-established dependence of cancer cells on their microenvironment suggests that targeting the non-cancer components of the TME could serve as a foundation for developing novel therapeutic approaches 39,40. Neutrophils, the most functionally abundant innate immune cells and key members of the TME, provide an important function in regulating cancer progression and therapeutic vulnerability 40. Neutrophils are actively involved in ongoing interactions between cancer cells, mesenchymal cells, and other immune cells, which may contribute to cancer-promoting effects 41. For example, tumor cell-secreted protease cathepsin C (CTSC) could recruit neutrophils to form neutrophil extracellular traps (NETs), thereby promoting breast-to-lung metastasis 42. Neutrophils are induced to accumulate neutral lipids upon interaction with resident mesenchymal cells in the premetastatic lung 43. Tumor-induced neutrophils acquire the ability to suppress cytotoxic T lymphocytes, which restricts the establishment and progression of BRCA metastases 44,45. In addition, the tumor-derived cytokine Chi3l1 induces the formation of NETs, which facilitates T-cell exclusion in triple-negative breast cancer 46.

The aforementioned functions of TANs underscore their therapeutic potential in the context of cancer. Indeed, various therapeutic approaches targeting TANs have demonstrated efficacy against BRCA. For example, pharmacological inhibition of the leukotriene-generating enzyme arachidonate 5-lipoxygenase (Alox5), which is responsible for generating leukotrienes, effectively eliminates neutrophil pro-metastatic activity and subsequently reduces metastasis 47. Targeting CTSC with compound AZD7986 effectively suppresses the recruitment of neutrophils and lung metastasis of BRCA 42. Neutrophil-specific deletion of genes encoding ATGL or ATGL inhibitory factors resulted in alterations to neutrophil lipid profiles and BRCA lung metastasis in mice 43. However, due to the underappreciation of TAN-related prognostic signatures in BRCA, as well as the lack of integration of single-cell and bulk transcriptome data, there remains a limited availability of effective biomarkers for developing treatments targeting TANs. Therefore, it is crucial to further investigate and identify novel valuable targets specific to TANs that have significance in evaluating BRCA prognosis and treatment response.

Our analysis showed that tumor-infiltrating TAN abundance was suggestive of poor BRCA prognosis, highlighting the importance of studying TANRGs. By integrating scRNA-seq data and bulk transcriptomic WGCNA, we have developed a TANRS for BRCA. This TANRS effectively risk-stratified BRCA patients, where patients with high TANRS had a poorer clinical prognosis, significant immunosuppression in TME, and poorer response to treatment. Thus, our screening strategies have identified prognostic genes associated with TANs and established novel TAN-related predictive biomarkers, providing new regulatory targets and insights for managing and treating BRCA.

A significant achievement of this research is the identification of novel therapeutic targets associated with TANs in BRCA. The nine prognostic TANRGs, including TAGLN2, IGF2R, LAMP2, TBL1X, ASAP1, DENND5A, SNRK, BCL3, and CEBPD, were determined as promising TAN-related biomarkers correlated with the prognosis, tumor immunity and treatment outcomes in BRCA patients. Some of these TANRGs have been described as regulating the progression and prognosis of BRCA or other tumors before, which partly verifies the reliability of the TANRGs identified in this study. For example, the loss of Transgelin-2 (TAGLN2), a 22 kDa actin-binding protein, resulted in significant deficiencies in the abilities of DCs to migrate to draining lymph nodes (LNs) and stimulate T cells to generate antigen-specific T cell clones. These alterations were correlated with an inability to suppress tumor growth and metastasis of melanoma cells in mice 48. ArfGAP with SH3 domain, ankyrin repeats, and PH domain 1 (ASAP1) is associated with poor survival in breast cancer, while it has also been reported that the loss of ASAP1 in luminal breast cancer accelerates tumorigenesis and promotes metastasis by activating AKT 49. The DENND5A variant promotes familial cutaneous melanoma by accelerating the degradation of pre-melanosome proteins and tyrosinase through lysosomes 50. BLC3 is recognized as an independent risk factor in BRCA, and evidence demonstrating its well-tolerated inhibition in animal models underscores its potential as a promising therapeutic target for these clinical scenarios 51. The key prognostic TANRGs in our study, SNRK, have been reported to mediate DNA damage and induce nuclear morphological changes through actin depolymerization 52. Additionally, SNRK contributes to angiotensin II-induced renal inflammation and fibrosis by coacting with NF-kappaB/p65 53. However, there is limited research on the role of SNRK in BRCA; therefore, further investigation is warranted to explore its potential therapeutic value. Our study raises the novel idea that SNRK is promising as a novel TAN-related target capable of suggesting BRCA prognosis.

Our study also has certain limitations. Immunotherapy has demonstrated enhanced efficacy in cancer treatment, particularly when combined with other therapeutic modalities. Therefore, further evaluation is warranted to assess the capability of the TANRS in predicting immunotherapy sensitivity and even other combined therapies. In addition, further exploration is warranted to assess the application value of this prognostic model in clinical practice. Moreover, comprehensive experimental validation is required to elucidate the function and mechanism of the TANRGs screened in this study.

## Conclusion

This TAN-related risk signature represents a reliable method for prognosis prediction, immune characterization, and therapeutic response assessment in BRCA patients. This study provides valuable insights for studying and innovating molecular mechanisms and clinical intervention strategies for BRCA.

## Supplementary Material

Supplementary figures.
