# Supplementary material for: A Novel Tumor-Associated Neutrophil-Related Risk Signature Based on Single-Cell and Bulk RNA-Sequencing Analyses Predicts the Prognosis and Immune Landscape of Breast Cancer
Source: J Cancer. 2024 Sep 3;15(17):5655–71. doi: 10.7150/jca.100338 (PMC11414621; doi:10.7150/jca.100338)
Supplement: Supplementary file 1 — Supplementary figures. [file jcav15p5655s1.pdf]

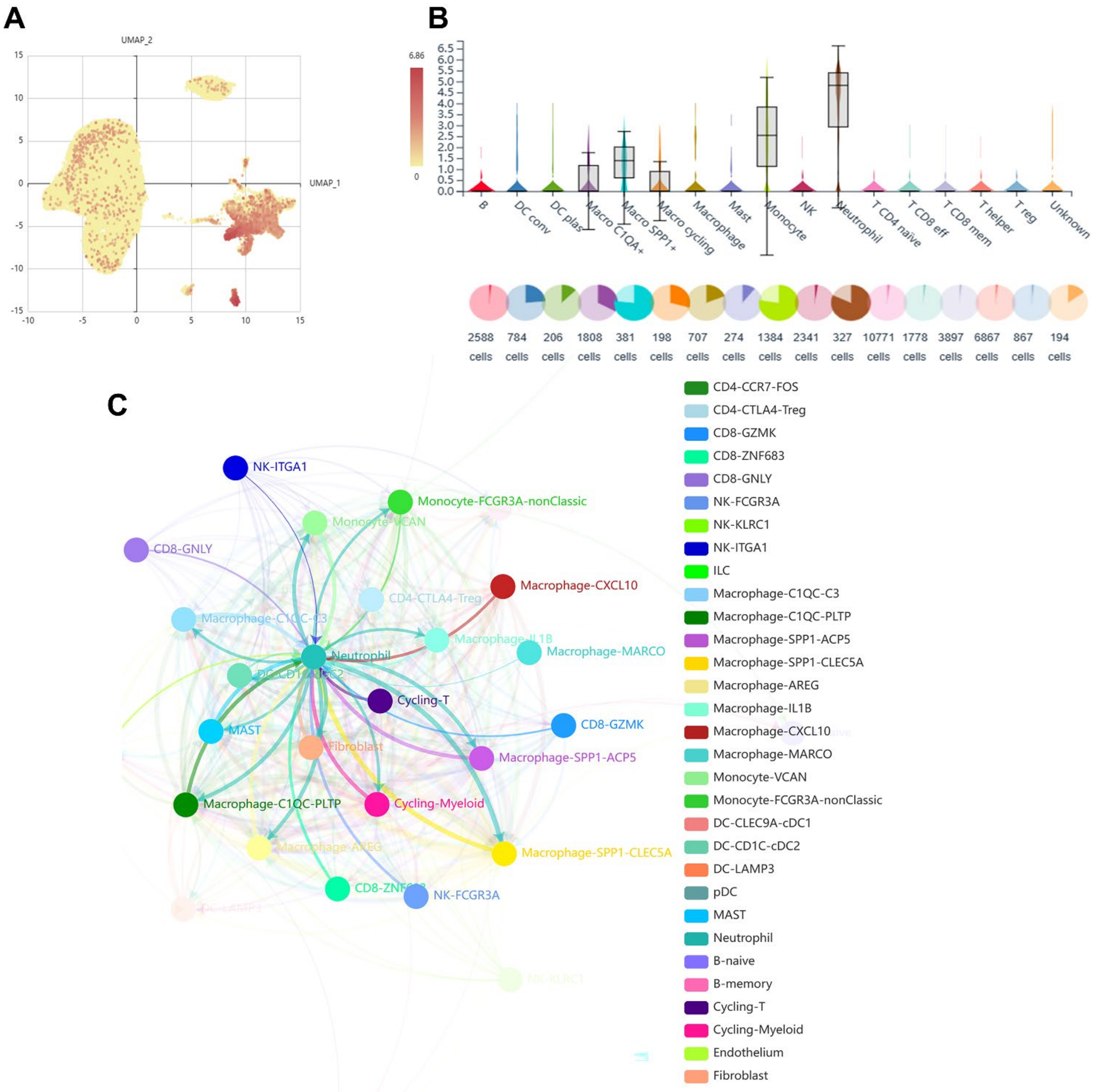

Figure S1. Identification of marker genes of TANs using scRNA-seq dataset GSE114725. (A, B) The expression of neutrophil marker gene S100A9 in different cell populations. (C) Cell communication network.

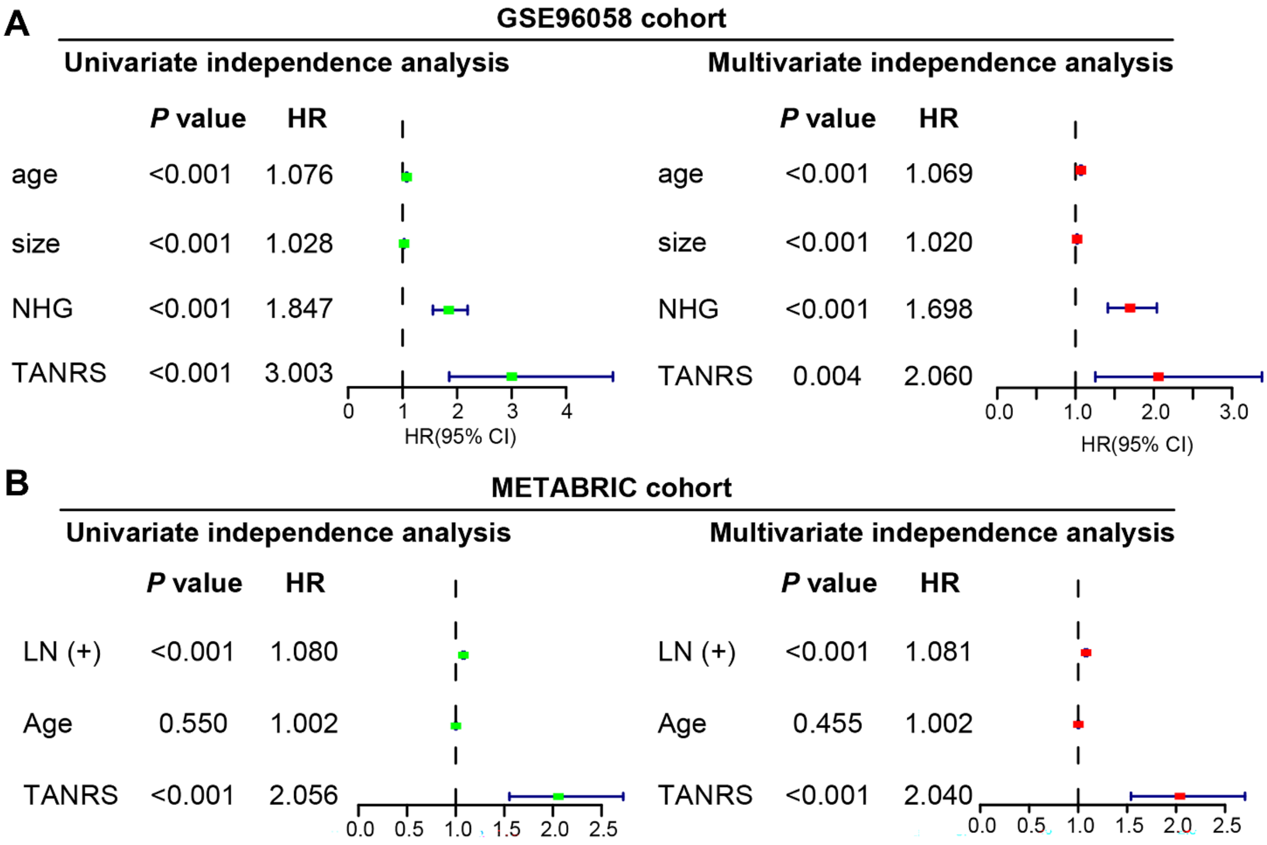

Figure S2. Validation of the independent prognostic potential of the TANRS in GSE96058 (A) and METABRIC (B) cohorts.

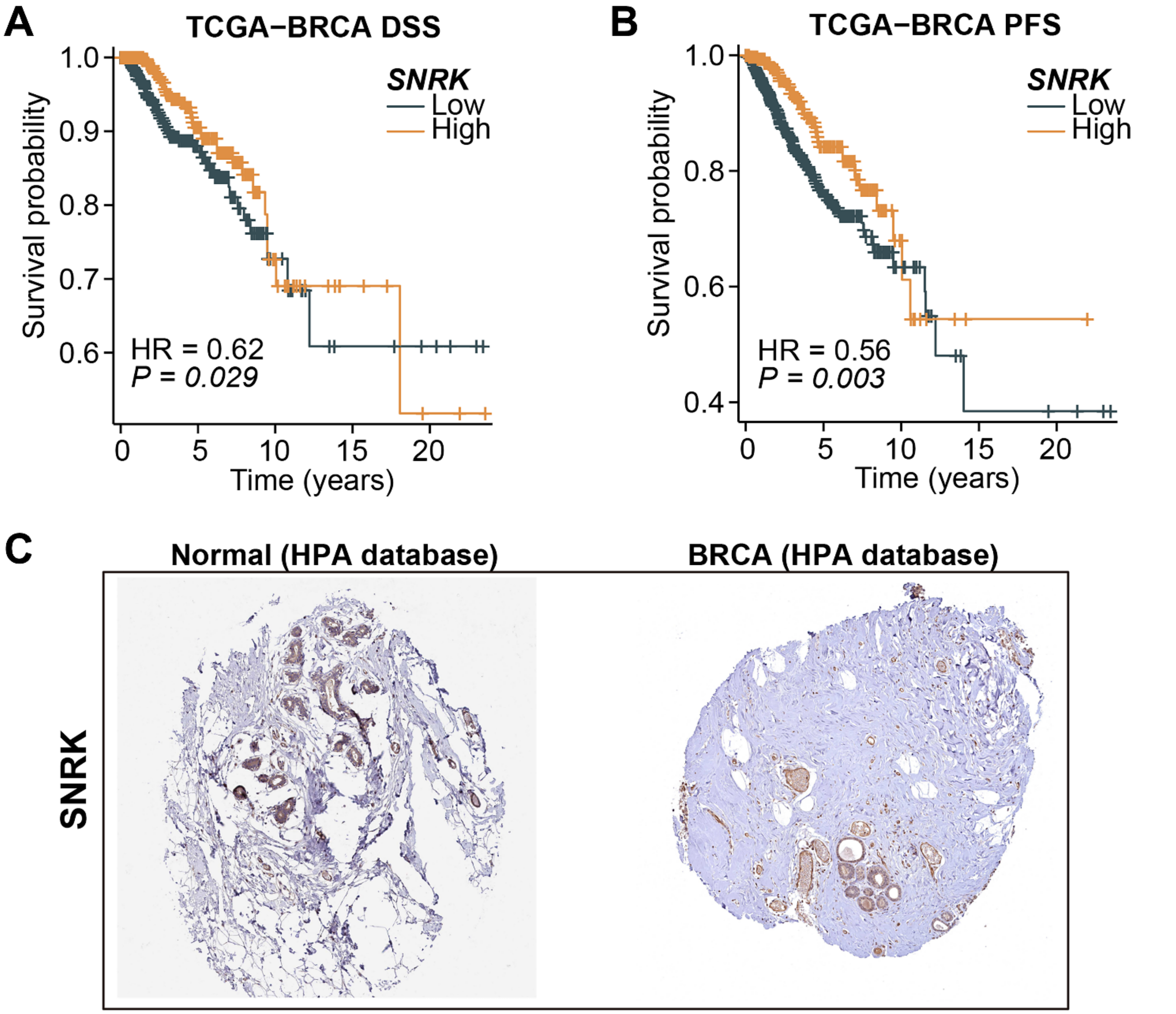

Figure S3. SNRK as a key TANRG in BRCA. (A, B) Prognostic significance of SNRK expression in BRCA. (C) IHC analysis of SNRK level in normal and BRCA samples based on the HPA database.
